# Sorption, Structure and Dynamics of CO_2_ and Ethane in Silicalite at High Pressure: A Combined Monte Carlo and Molecular Dynamics Simulation Study

**DOI:** 10.3390/molecules24010099

**Published:** 2018-12-28

**Authors:** Siddharth Gautam, Tingting Liu, David Cole

**Affiliations:** School of Earth Sciences, The Ohio State University, Columbus, OH 43210, USA; liu.2189@osu.edu (T.L.); cole.618@osu.edu (D.C.)

**Keywords:** sorption, molecular dynamics, Monte Carlo, CO_2_, ethane, silicalite

## Abstract

Silicalite is an important nanoporous material that finds applications in several industries, including gas separation and catalysis. While the sorption, structure, and dynamics of several molecules confined in the pores of silicalite have been reported, most of these studies have been restricted to low pressures. Here we report a comparative study of sorption, structure, and dynamics of CO_2_ and ethane in silicalite at high pressures (up to 100 bar) using a combination of Monte Carlo (MC) and molecular dynamics (MD) simulations. The behavior of the two fluids is studied in terms of the simulated sorption isotherms, the positional and orientational distribution of sorbed molecules in silicalite, and their translational diffusion, vibrational spectra, and rotational motion. Both CO_2_ and ethane are found to exhibit orientational ordering in silicalite pores; however, at high pressures, while CO_2_ prefers to reside in the channel intersections, ethane molecules reside mostly in the sinusoidal channels. While CO_2_ exhibits a higher self-diffusion coefficient than ethane at low pressures, at high pressures, it becomes slower than ethane. Both CO_2_ and ethane exhibit rotational motion at two time scales. At both time scales, the rotational motion of ethane is faster. The differences observed here in the behavior of CO_2_ and ethane in silicalite pores can be seen as a consequence of an interplay of the kinetic diameter of the two molecules and the quadrupole moment of CO_2_.

## 1. Introduction

The behavior of molecules is strongly determined by their environment, and is different if they are surrounded by molecules of the same species or of a different species. The latter scenario occurs, for example, when a fluid is confined in a porous media, or is adsorbed on the surface of a substrate. The specific interaction between the fluid molecules and the confining substrate alters the behavior of the fluid. As most of the interesting chemical reactions occur at interfaces between two species, the behavior of molecules under confinement or at an interface in general is an interesting topic of research [1,2,3,4,5]. An important component that determines the interaction between molecules in general is the distribution of the electrostatic charge. In some molecules such as water, an asymmetrical distribution of charge gives rise to a permanent dipole moment. The distribution of charge in a molecule plays an important role in determining the dynamical behavior. For example, it has been found that for molecules of similar shapes and sizes, both the translational and the rotational motions are severely affected by an asymmetry in the charge distribution [6,7]. The motion of acetonitrile, acetaldehyde, and acetone, all with a non-zero dipole moment was found to be severely restricted, whereas that of propane with no dipole moment was found to be less restricted. In the studies mentioned above, the motion of these molecules confined in all silica analogues of ZSM-5–silicalite was studied. Silicalite imposes strict geometrical confinements on the sorbed molecules, due to its small channel-like pores, which are, on average, 0.55 nm in diameter. With the kinetic diameters of the sorbed molecules being of a size that is comparable to the pore diameter in these studies, an interplay between the effects of the dipole moment and the geometrical confinement on these molecules resulted in severely restricted motion for all molecules except for propane.

CO_2_ is a linear molecule with no dipole moment, but with a finite quadrupole moment. This quadrupole moment of CO_2_ gives rise to interesting phenomena. For example, it has been suggested that due to a stronger interaction with quadrupolar CO_2_, this molecule replaces hydrocarbons from silica pore walls. This has been shown to give rise to an enhancement in the diffusivity of confined hydrocarbon by the addition of CO_2_ in several studies [8,9,10]. Juxtaposing the quadrupolar nature of CO_2_ with the effects of dipole moments on the motion of molecules in silicalite reported in [6,7], a question can be raised whether the quadrupolar nature of CO_2_ can also result in a severely restricted motion of CO_2_ in silicalite. To address this, a detailed study of CO_2_ dynamics in silicalite is needed. The sorption and mobility of gases such as CO_2_ and ethane inside ZSM-5 has been studied by several authors [11,12,13,14,15]. More recently, mixtures containing CO_2_ in silicalite have also been studied [16,17,18]. Moisture-driven CO_2_ sorption has also been studied. For example, Shi et al. have reported the effects of moisture on the sorption of CO_2_ in nano-structured sorbents [19,20]. However, most of these studies were limited to lower loadings corresponding to pressures of a few tens of bar. Studies at higher pressures are lacking. In particular, there are very few studies addressing molecular motion under confinement at supercritical densities. The effects of high densities encountered in nanoporous engineered and natural materials are thus underrepresented in the literature.

In molecular level computer simulations, the force field used is an important parameter that can determine the course of the simulation. These force-fields are usually obtained by fitting some experimental data. For small hydrocarbons and CO_2_, the TraPPE (transferable potentials for phase equilibria) force field developed by Martin and Siepmann [21] is commonly used. Alkanes are generally modeled in united atom (UA) formalism in this force field, and an ethane molecule is modeled as a dumbbell-type diatomic molecule with two CH_3_ pseudo-atoms. An ethane molecule modeled in the TraPPE-UA force field is thus similar to the linear CO_2_ molecule, although with a kinetic diameter of 0.44 nm [22], the ethane molecule is slightly bigger than the CO_2_ molecule with a kinetic diameter of 0.33 nm [23] (the kinetic diameter is a measure of the sphere of influence of a molecule, and it represents the size of a molecule in reference to intermolecular collision events). A comparative study of CO_2_ and ethane can reveal the role of the quadrupole moment in the sorption and dynamics of these two molecules inside silicalite. Here, we report grand canonical Monte Carlo (GCMC) and molecular dynamics (MD) studies on the sorption, structure, and dynamics of ethane and CO_2_ in silicalite at higher loadings corresponding to a partial pressure of up to 100 bar. To achieve supercritical densities for both CO_2_ as well as ethane, this study was carried out at a temperature of 308 K. At this temperature, the bulk state of CO_2_ and ethane becomes supercritical at 46 bar and 77 bar, respectively [24].

## 2. Results

### 2.1. Sorption Isotherms of CO_2_ and Ethane in Silicalite

**Figure 1:**
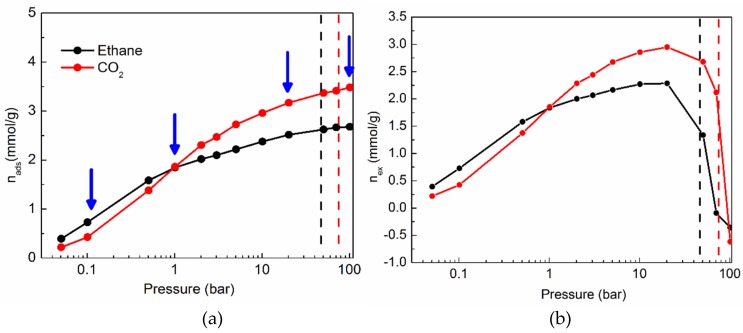
Sorption isotherms of ethane (black) and CO_2_ (red) in silicalite at 308 K. (**a**) Absolute sorption; (**b**) Excess sorption. Symbols denote the data obtained from the GCMC simulations, while the solid lines serve as guides for the eye. The supercritical pressures of bulk ethane and CO_2_ are marked by black and red dashed lines, respectively, in both panels. Blue arrows indicate the gas loadings at which MD simulations were carried out for both ethane and CO_2_ in silicalite.

Figure 1a shows the sorption isotherms of ethane and CO_2_ in silicalite at 308 K. At pressures below atmospheric pressure (1 bar), ethane is adsorbed in silicalite more favorably. However, at higher pressures, the sorption of CO_2_ is more favorable. The supercritical pressures of bulk ethane and CO_2_ are marked in the figure by using vertical dashed lines of black and red colors, respectively. The density of a fluid adsorbed in a nanoporous material at a given temperature and pressure condition is generally higher than the bulk density at those conditions. It is therefore customary to express the sorption isotherms in terms of the excess density (*n_ex_*) of the sorbed fluid over the corresponding bulk density [25]. The relation between the sorbed density (*n_ads_*) and the excess density can be expressed as:

*n_ex_* = *n_ads_* − *V_p_ρ_b_*/*M* (1)

where *V_p_* is the volume of the pores accessible to the fluid, *M* is its molar mass, and *ρ_b_* is the bulk density of the fluid at the corresponding temperature and pressure conditions. The quantity *n_ads_* is obtained directly from the GCMC simulation. First et al. [26] used an automated approach to characterize several zeolitic frameworks, and obtained pore volumes. For our calculations of the excess sorption, we used their estimate of the accessible pore volume in silicalite. The values of the bulk density of several fluids including CO_2_ and ethane are available on the NIST web-book [24]. Using these quantities, the excess sorption isotherms of ethane and CO_2_ obtained at 308 K are shown in Figure 1b. Both ethane and CO_2_ show a region of enrichment in the pores, up to a pressure of 20 bar, followed by depletion. In what follows, we shall discuss the results from MD simulations for ethane and CO_2_ in silicalite, carried out on loadings corresponding to three subcritical and one supercritical pressure (0.1, 1, 20 and 100 bar). These pressures are also marked in Figure 1a.

### 2.2. Structure of Ethane and CO_2_ in Silicalite

#### 2.2.1. Distribution of the Molecules in Different Pores

**Figure 2:**
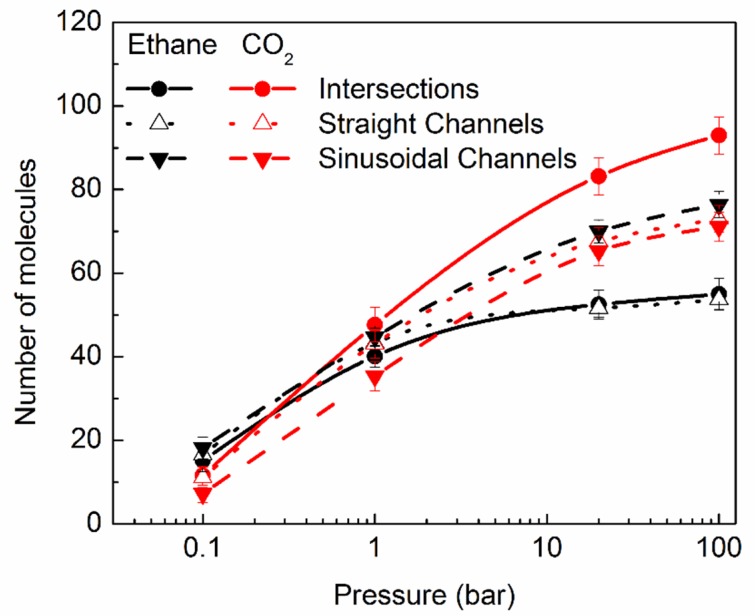
Distribution of the sorbed molecules among the three pore types of silicalite: intersections (circles), straight channels (open triangles), and sinusoidal channels (solid triangles). Black symbols represent the data for ethane, while red symbols show data for CO_2_.

Silicalite has a complex pore structure, with pores in the form of straight and sinusoidal channels, with their intersections exhibiting somewhat larger dimensions then the channels themselves. While the straight channels are oriented along the Cartesian Y direction, the sinusoidal channels lie in the X–Z plane. Due to the slightly different shapes of these pores, we explored which of these three pore types affords hosting of the guest molecules more readily. To probe this, we obtained the distribution of the molecules amongst these three pore types, in all simulations. These distributions are shown in Figure 2 for both ethane and CO_2_, with black and red symbols, respectively. At lower pressures, the difference in the number of ethane molecules is similar in all channels, within one standard deviation. However, at higher pressures, ethane molecules show a clear preference for residing in the sinusoidal channels. This could be because a molecule of large enough kinetic diameter might find it difficult to exit out of sinusoidal channels, due to its tortuosity. On the other hand, CO_2_ has a smaller kinetic diameter, resulting in an increased ability to move out of these channels, and therefore it shows no preference to reside in them, rather preferring intersections.

#### 2.2.2. Orientational Distribution of Molecules in Silicalite and Anisotropy

**Figure 3:**
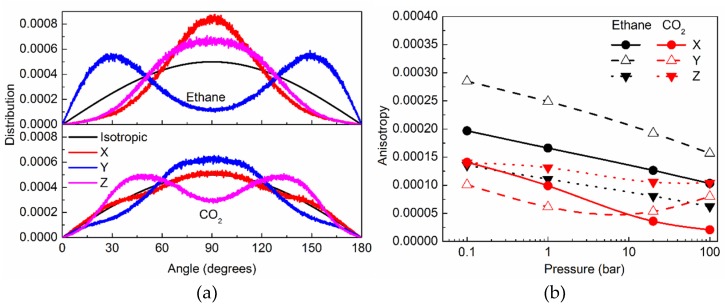
Orientational structure of the sorbed molecules in the straight channels of silicalite: (**a**) Orientational distribution with respect to the Cartesian axes X (red), Y (blue), and Z (magenta). Distribution expected in the case of an isotropic system is also included for reference as a black curve; (**b**) Anisotropy in the orientational distribution along different directions X (solid circles), Y (open triangles), and Z (solid triangles) as a function of the pressure of ethane (black) and CO_2_ (red). Lines in panel (b) serve as a guide to the eye.

In a matrix like silicalite, that puts severe geometrical constraints on the sorbed molecules, both positional as well orientational ordering of molecules can be expected. Indeed, orientational ordering has been observed for several molecules that have been sorbed in silicalite [6,15,27]. Orientational ordering is observed overall for both ethane and CO_2_ in silicalite (See Supplementary Materials, Figure S1). Due to the tortuous shape of the sinusoidal channels a wide distribution of orientations can be expected for confined molecules. Likewise, at channel intersections, the geometrical restriction is slightly relaxed, due to a larger pore volume and ellipsoidal shape; thus, orientational ordering of sorbed molecules is expected to be less constrained, resulting in relatively more disorder. In straight channels that are parallel to the Cartesian Y axis, the ordering is relatively stronger, and the distribution is easier to interpret. Therefore, in Figure 3a, we show the orientational distribution function (ODF) of ethane and CO_2_ at 100 bar in the straight channels of silicalite, with respect to the three Cartesian directions. In absence of a preferred orientation, the distribution is expected to be isotropic, and the corresponding ODF is also included in Figure 3a for comparison. Any deviation from the isotropic ODF signifies a preferred orientation. Clearly, both ethane and CO_2_ show deviations from isotropic behavior. However, the preferred directions in the two cases are different. While ethane prefers to lie mostly along the channel axis (with the Y-direction showing stronger than isotropic distribution at lower (closer to 0°) and higher (closer to 180°) angles), CO_2_ prefers to lie perpendicular to the channel axis (with the Y-direction ODF peaking at 90°, and being weaker at lower and higher angles, compared to isotropic ODF).

The extent of the deviation in the orientational distribution with respect to the isotropic case can be used to quantify the anisotropy in the orientational structure of the sorbed molecules. Following previous studies [6,15], we define the degree of anisotropy (*ϕ*) in the orientational structure as:

*ϕ* = [(1/*N*)Σ(*g_iso_*(*θ*) − *g_s_*(*θ*))^2^]^1/2^ (2)

where *g_i_*(*θ*) is the orientational distribution function, the subscript *i* denotes the case of isotropic distribution (iso) or the silicalite (s), and the sum is taken over *N* = 3150 different values of *θ* over which the ODF were calculated. The degree of anisotropy that is defined in this manner is shown in Figure 3b for the three Cartesian directions for ethane and CO_2_ in straight channels of silicalite at all pressures. For ethane, the variation of *ϕ* with pressure shows a consistent trend in all directions; namely, *ϕ* decreases with an increase in pressure. For CO_2_, however, the trend is different in different directions, with *ϕ* decreasing at higher pressures monotonously in the Z and X directions, while the variation in the Y direction is non-monotonous.

### 2.3. Dynamics

#### 2.3.1. Translational Motion and its Anisotropy

**Figure 4:**
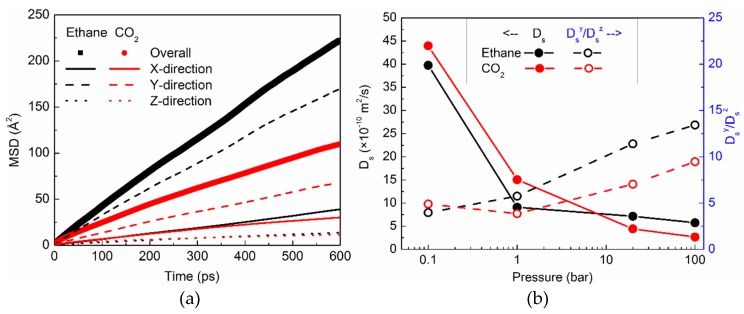
(**a**) *MSD* of ethane (black) and CO_2_ (red) in silicalite in different Cartesian directions and overall. (**b**) The self-diffusion coefficient of ethane (solid black symbols) and CO_2_ (solid red symbols). Anisotropy in translational motion (*D_s_^Y^*/*D_s_^Z^*) of ethane (open black symbols) and CO_2_ (open red symbols). The Y-axis labels for the self-diffusion coefficient can be read on the left, while those for anisotropy appear on the right in blue.

Translational motion of ethane and CO_2_ in silicalite is studied by using the mean squared displacement (*MSD*) of the sorbed molecules. In an anisotropic matrix such as silicalite, *MSD* in different directions can be expected to be different. In Figure 4a, we show the *MSD* of ethane and CO_2_ in the three Cartesian directions X, Y, and Z, along with the overall *MSD* at 100 bar. At this pressure, ethane can be seen to exhibit consistently higher *MSD* than CO_2_ in all directions. This is in spite of the smaller kinetic diameter of CO_2_, as compared to ethane. A significant degree of anisotropy can be observed in the *MSD*, with the Y direction showing the highest *MSD* for both ethane and CO_2_, whereas the Z direction shows the smallest *MSD*. This is because of the straight channels which because of their geometry, facilitate the motion of the sorbed molecules that lie along the Cartesian Y directuion, whereas the sinusoidal channels with their tortuous shape inhibiting motion, lie in the X–Z plane. *MSD* is a measure of the self-diffusive motion of molecules and at long times, it can be used to obtain the self-diffusion coefficient (*D_s_*) using the Einstein relation:

*D_s_* = *MSD*/(2*n_d_t*) (3)

where *n_d_* is the number of degrees of freedom, and *t* is time. For consistency, we used the slope of *MSD* vs. *t* curve between *t* = 400 ps and *t* = 600 ps, to obtain the self-diffusion coefficient in all cases. In Figure 4b, we show the self-diffusion coefficient of ethane and CO_2_ (solid black and red symbols respectively) as a function of pressure. The variation of *D_s_* with pressure is similar for both ethane and CO_2_. *D_s_* decreases at higher pressure, as can be expected, due to the crowding of molecules at higher pressures. At lower pressures, ethane exhibits slower diffusion compared to CO_2_, whereas at higher pressures, ethane becomes relatively faster. This could be because of the relatively lower amounts of ethane sorbed in silicalite at higher pressures. In Figure 4b, we also show the ratio of self-diffusivity in the directions Y and Z (*D_s_^Y^*/*D_s_^Z^*; open symbols) as a function of pressure. This ratio is a measure of the anisotropy in the translational diffusion. The anisotropy in translational diffusion is found to increase at higher pressures for both ethane and CO_2_. Further, the anisotropy at higher pressures is greater for ethane, compared to CO_2_. This could be a consequence of the slightly larger kinetic diameter of ethane, which makes it more difficult for ethane molecules to diffuse through sinusoidal channels (X–Z plane), compared to the straight channels.

Another important quantity that can be used to understand the translational motion of molecules is the velocity autocorrelation function (VACF, *C_v_*(*t*)).

*C_v_*(*t*) = <***v***(*t* + *t*_0_)·***v***(*t*_0_)> (4)

where ***v***(*t*) is the velocity of an atom at time *t*. The angular brackets denote an average over the number of atoms, as well as the time origins *t*_0_. The self-diffusion coefficient can also be obtained by integrating the *C_v_*(*t*) vs. *t* curve [28]. In addition, *C_v_*(*t*) can be used to calculate the vibrational spectrum (*I*(*ω*)) of the system, using the relation [29]:

*I*(*ω*) = *∫C_v_*(*t*)cos(*ωt*)*dt* (5)

**Figure 5:**
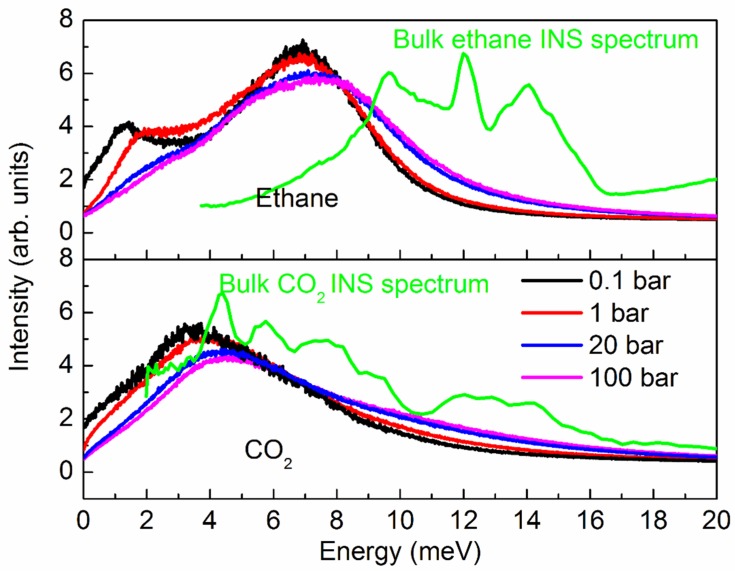
Vibrational spectra of ethane (top) and CO_2_ (bottom) in silicalite at different pressures. Also included in green are the spectra of bulk ethane [30] and CO_2_ [31], obtained in inelastic neutron scattering experiments and available at the ISIS INS database [32].

We point out that since both ethane and CO_2_ are modeled as rigid molecules here, the vibrational spectra obtained using the above equation will be limited to the intermolecular vibrational modes alone. In Figure 5, we show the vibrational spectra calculated from the VACF for ethane (top panel) and CO_2_ (bottom panel) at different pressures. While the spectra for ethane are bimodal at low pressures, the lower energy peak merges with the broad peak at higher pressures. CO_2_ on the other hand exhibits a single broad peak at all pressures. Further, a shift to higher energies can be observed in the spectra for both ethane and CO_2_ at higher pressures. A similar shift to higher energies has been observed in the vibrational spectrum of propane confined in MCM-41-S in an inelastic neutron scattering experimental study [29]. For comparison, the vibrational spectra of bulk ethane [30] and CO_2_ [31] obtained from inelastic neutron scattering experiments are also included. These spectra are taken from the ISIS INS database [32]. Although the spectra calculated from a simulation of rigid molecules is not expected to capture all the details of the experimental spectra, some salient features are reproduced. For example, the intermolecular vibrational modes of CO_2_ confined in silicalite peak at lower energies compared to ethane, a result that is qualitatively consistent with the experimental data on bulk ethane and CO_2_. Further, the calculated spectra of both ethane and CO_2_ look more disordered, as compared to the bulk spectra. A similar confinement-induced disorder was observed in the vibrational spectra of propane in MCM-41-S [29] obtained from INS experiments, as well as MD simulations.

#### 2.3.2. Rotational Motion

The preferred orientations seen in the ODF in Figure 2 for both ethane and CO_2_ suggest that the rotational motion of the sorbed molecules is restricted. The rotational motion of rigid molecules in general can be studied by following the orientation of a unit vector (***u***) that is attached to the molecule as a function of time [33,34]. In particular, one can follow the evolution of the first-order rotational correlation function (RCF, *C_R_*(*t*)), defined as:

*C_R_*(*t*) = <***u***(*t+t_0_*).***u***(*t_0_*)> (6)

**Figure 6:**
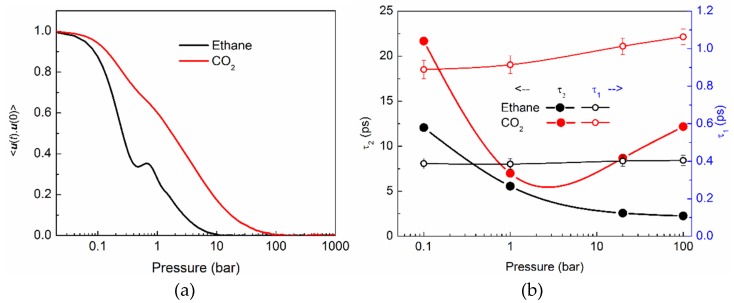
Rotational motion of ethane (black) and CO_2_ (red) in silicalite: (**a**) Rotational correlation function (RCF) at 100 bar. (**b**) Time scales of rotational motion obtained by fitting the two regimes of RCF with an exponential decay. In the first regime, τ_1_ was obtained by fitting the RCF between 0 and 0.2 ps. The Y-axis labels for τ_1_ can be read on the right in blue, while those for τ_2_ appear on the left.

In Figure 6a we show the RCF of ethane (black curve) and CO_2_ (red curve) at 100 bar. Compared to ethane, the RCF of CO_2_ takes longer to decay to zero, indicating a slower rotational motion of CO_2_ compared to ethane. Further, the RCF of both ethane and CO_2_ exhibit distinct behaviors at times that are shorter than a picosecond, and longer times. This facilitates dividing the RCF in two regimes of sub-picosecond times, and longer times. The boundary of these two regimes is marked clearly in case of ethane in the form of a kink. As mentioned in previous publications, this is a signature of vibrational motion [15,27]. No kink can be observed in the case of CO_2_ suggesting that the rotational motion of CO_2_ is different from the libration type of motion exhibited by ethane and other molecules [6,15,27]. CO_2_ has a kinetic diameter that is considerably smaller than the channel diameter of silicalite. Further, the interaction of the molecule with the pore walls is not strong enough to restrict it from rotating more freely inside the silicalite channels, tracing out the entire orientational space available.

To estimate the time scales of rotational motion, we fitted the RCF in the two regimes with exponential decay functions. The time scales in the two regimes of rotational motion, τ_1_ (shorter time scale regime, open symbols) and τ_2_ (longer time regime, solid symbols) are shown in Figure 6b for ethane (black) and CO_2_ (red) at different pressures. For consistency, all RCFs were fit between *t* = 0 and *t* = 0.2 ps; and *t* = 2 ps and *t* = 200 ps to obtain τ_1_ and τ_2_, respectively. The rotational motion of CO_2_ is slower than that of ethane in both regimes. Although the short time rotational motion of CO_2_ shows a monotonous decrease at higher pressures, the longer time rotation becomes faster initially, when pressure is increased to 1 bar, and for higher pressures, it slows down again. For ethane, the short time rotational motion is independent of pressure, while the longer time rotation becomes faster consistently with an increase in pressure.

## 3. Discussion

The sorption isotherms presented in Figure 1 are in very good agreement with those reported by Sun et al. [13] for ethane in silicalite at 308 K up to 3.43 bar, and CO_2_ in silicalite up to 1.37 bar. Ethane shows a stronger sorption at lower pressures, while CO_2_ is adsorbed more readily at higher pressures. In a study on CO_2_ sorption in metal organic frameworks at 298 K up to 35 bar, Walton et al. [35] found that a higher amount of CO_2_ is adsorbed in the metal organic framework, if electrostatic interactions are accounted for in the simulation. Conversely, lower amounts of CO_2_ were found to be adsorbed if the electrostatic interactions were switched off. The higher sorption of CO_2_ is thus due to the electrostatic interactions between the quadrupole moment of CO_2_ and the pore surface.

Both ethane and CO_2_ exhibit type I isotherms up to 100 bar. It is interesting to note that all three types of pores in silicalite intersections, straight channels, and sinusoidal channels are accessible to both ethane, as well as CO_2_ at all pressures, as shown in Figure 2. Even at lower pressures not investigated with MD, GCMC data show that the molecules start to populate all three types of pores (see Supplementary Materials, Figure S2). This is in contrast to the case of branched alkanes, e.g., iso-butane, that populate only the intersections at lower pressures, resulting in a type IV isotherm, as observed by Vlugt et al. [36]. This is because of the relatively smaller kinetic diameter of ethane and CO_2_, compared to iso-butane. It is natural that CO_2_ shows a clear preference for intersections at higher pressure. This is because the intersections offer a marginally larger ellipsoidal free space, compared to the straight and sinusoidal channels. However, ethane molecules at higher pressures prefer to stay in sinusoidal channels, although at lower pressures, this preference is not very clear. We note that in our previous study of ethane in silicalite, the highest loading investigated (eight molecules per unit cell) corresponded to a pressure of slightly less than 1 bar. At that pressure, the error bars in Figure 2 make no conclusion about the preference for pore type difficult.

The orientational order exhibited by both ethane and CO_2_ (Figure 3) is consistent with that exhibited by several other molecules confined in silicalite [6,15,27]. This behavior results from a strict geometrical restriction imposed by the narrow silicalite pores. Further, the preferred orientation of CO_2_ in straight channels, perpendicular to the channel direction, is a consequence of the partial charges on the extremities of the molecule. The electrostatic interactions between the extremities of CO_2_ molecule and the pore wall make the molecule lie perpendicular to the channel, whereas in the absence of such partial charges, ethane molecules prefer a parallel orientation. This has important consequences for the motion of these molecules. Being linear molecules, it can be expected that the motions of both these molecules along their molecular axes will be easier. Indeed, for ethane, it was found that the *MSD* parallel to the molecular axis is considerably higher than that which is perpendicular to the axis (see Supplementary Materials, Figure S3). However, for CO_2_, no considerable difference was observed for *MSD* that was parallel or perpendicular to the molecular axis. Although, in a free environment, the motion of a CO_2_ molecule along the molecular axis would be easier, in case of silicalite, no free space is available in that direction, as the molecule lies perpendicular to the channel axis.

At lower pressures, CO_2_ diffuses faster than ethane. However, at those pressures, the amount of CO_2_ adsorbed in silicalite is lower than that of ethane. Thus, crowding effects on the translation diffusion for ethane are stronger. However, at higher pressures, the relative amount of CO_2_ is larger than ethane, and hence the diffusivity of CO_2_ is slower. The variation of self-diffusivity can be viewed as the interplay of the kinetic diameters and crowding effects. The rotational motion of CO_2_ in silicalite is slower than ethane, in spite of a slightly smaller kinetic diameter. While for ethane, the rotational motion gets consistently faster at higher pressures, for CO_2_ the variation is non-monotonous. In a previous work on ethane rotation in silicalite [15], we observed a correlation between the variation of rotational motion and anisotropy in the ODF. We note the same correlation here in the case of ethane, wherein as the anisotropy decreases at higher pressure, the rotational motion becomes faster. A similar correlation can also be seen between the rotational motion of CO_2_ and anisotropy in ODF in the Y direction.

Although the behavior of both ethane and CO_2_ in silicalite at pressures corresponding to bulk supercritical pressures is quantitatively different from that at lower pressures, no major qualitative changes were observed at this pressure (100 bar) compared to the lower pressures corresponding to bulk sub-critical pressures.

In the absence of electrostatic interactions, CO_2_, which is a smaller molecule, can be expected to experience relatively less restriction to motion by the confining media. However, due to the quadrupole moment of CO_2_ that appears as partial charges on the C and O atoms in the TraPPE model used here, CO_2_ is not only adsorbed in silicalite to a greater extent, its motion is also suppressed more, compared to ethane. On the other hand, the suppression in the mobility of CO_2_ is not as drastic as what is observed in the case of molecules with dipole moments that barely move inside silicalite [6,7]. Thus, although not as strongly as the dipole moment of other molecule, the quadrupole moment of CO_2_ does affect its sorption, structure, and dynamics in silicalite.

## 4. Materials and Methods

In this work we used Grand Canonical Monte Carlo (GCMC) simulations to obtain the sorption isotherms of ethane and CO_2_ in silicalite, and Molecular Dynamics (MD) simulations to study their structural and dynamical properties. While GCMC simulations were carried out using DL_Monte [37], MD simulations were carried out using DL_Poly [38]. Both these packages have been developed by CCP Forge, and they are available free of charge. DL_Monte has been shown to have a computational performance that is comparable to several other Monte Carlo codes [39]. Besides, having commonalities with DL_Poly, it was a natural choice to complement the MD simulations carried out using DL_Poly.

A model ZSM-5 pore network was built by making use of the co-ordinates provided by Koningsveld et al. [40]. To start with, two molecules each of CO_2_ or ethane were loaded in the straight channels of ZSM-5. GCMC simulations were then carried out on the simulation cell thus obtained. During the simulation, the sorbed molecules, i.e., ethane or CO_2_, could be inserted, deleted, translated, or rotated, while all silicalite atoms were kept rigid. All simulations were carried out using a fixed gas partial pressure at 308 K. This is done by using the following selection procedures for the insertion (*P_i_*) or deletion (*P_d_*) of a molecule.

*P_i_* = *min*{1,(*βVP*)*exp*(−*β*Δ*U*)/(*N* + 1)} (7)

*P_d_* = *min*{1,(*N*/*βVP*)*exp*(−*β*Δ*U*)} (8)

where *V* is the simulation cell volume, *P* is the partial pressure of the gas, *U* represents the potential energy, *N* is the number of molecules and *β* = *k_B_T*.

To start with, GCMC simulations were carried out at the lowest pressure of 0.05 bar. After this, successive simulations were carried out by sequentially increasing the pressure. The final configurations of each simulation were used as the initial configurations for the next simulation. At lower pressures, 2 million Monte Carlo steps were enough to obtain statistically meaningful configurations. Of these, the first 500,000 steps were discarded to account for equilibrium. At higher pressures, 5 million steps were used, out of which, the first 150,000 were equilibration steps. Coordinates were sampled every 10,000 steps. During the simulation, the translation, rotation, insertion, and deletion of ethane or CO_2_ were undertaken with a probability of 0.25, 0.25, 0.5, and 0.5 respectively. A potential cut-off of 1.4 nm, as suggested for TraPPE-UA force field was used. The number of molecules of ethane or CO_2_ in the simulation cell averaged over the entire production run, discarding the equilibration steps, were used to obtain the adsorption isotherms as a function of pressure.

Having obtained the adsorption isotherms, four pressures of interest (0.1, 1, 20, and 100 bar) were selected to further investigate the structures and dynamics of ethane and CO_2_ in silicalite, using MD simulations. The outputs from the MC simulations were used as the inputs for the MD simulations in all cases. MD simulations were carried out in NVT ensemble, using a Nose-Hoover thermostat. A total of eight simulations, four simulations corresponding to the selected pressure each for ethane and CO_2_ in silicalite, were carried out by using a simulation time step of 1 fs. In each simulation, the system was allowed to equilibrate at 308 K for 0.5 ns. After this, trajectories were recorded at each 0.02 ps for a duration of 1.5 ns. Various properties of interest were then calculated from these trajectories. The achievement of equilibrium was confirmed when the energy and temperature values showed no clear monotonous increase/decrease in time, and the variations were less than 5%.

## 5. Conclusions

We studied the sorption, structure, and dynamics of ethane and CO_2_ in silicalite—two similar molecules without and with a quadrupole moment. The quadrupole moment of CO_2_ plays an important role in all of the properties investigated here. Combining the information obtained here with some previous studies, we can conclude that the effects of electrostatic interactions on the properties of a confined fluid in a nanoporous medium are strongest for molecules with a net dipole moment, and become progressively milder for molecules with a quadrupole moment and no polarity.

## Supplementary Materials

The Supplementary Materials are available online. Figure S1: Orientational distribution functions of ethane and CO_2_ in all pores of silicalite, Figure S2: Distribution of ethane and CO_2_ in straight and sinusoidal channels, and their intersections at 0.05 bar; Figure S3: *MSD* of ethane and CO_2_ parallel and perpendicular to the molecular axis at 100 bar.
