# Supplementary material for: Sorption, Structure and Dynamics of CO2 and Ethane in Silicalite at High Pressure: A Combined Monte Carlo and Molecular Dynamics Simulation Study
Source: Molecules. 2018 Dec 28;24(1):99. doi: 10.3390/molecules24010099 (PMC6337235; doi:10.3390/molecules24010099)
Supplement: Supplementary file 1 [file molecules-24-00099-s001.pdf]

Sorption, Structure and Dynamics of CO_2 and Ethane in Silicalite at High Pressure: A Combined Monte Carlo and Molecular Dynamics Simulation Study

Siddharth Gautam *, Tingting Liu and David Cole

School of Earth Sciences, The Ohio State University, Columbus, OH 43210, USA;
liu.2189@osu.edu (T.L.); cole.618@osu.edu (D.C.)
* Correspondence: gautam.25@osu.edu; Tel.: +01-614-292-7365

Supplementary Materials

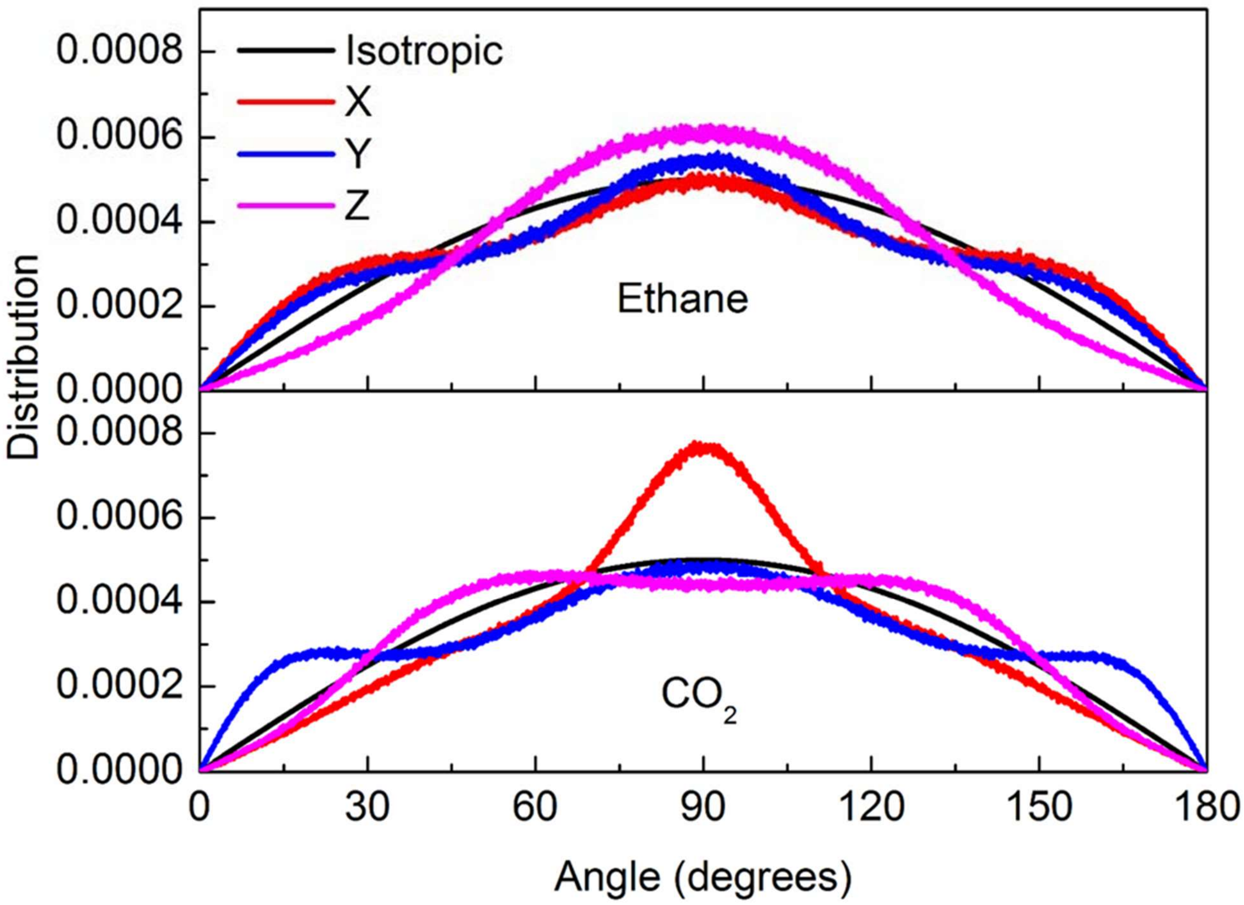

Figure S1. Orientational distribution functions of ethane and CO_2 in all pores of silicalitewith respect to the Cartesian axes X (red), Y (blue) and Z (magenta). Distribution expected in case of an isotropic system is also included for reference as a black curve.

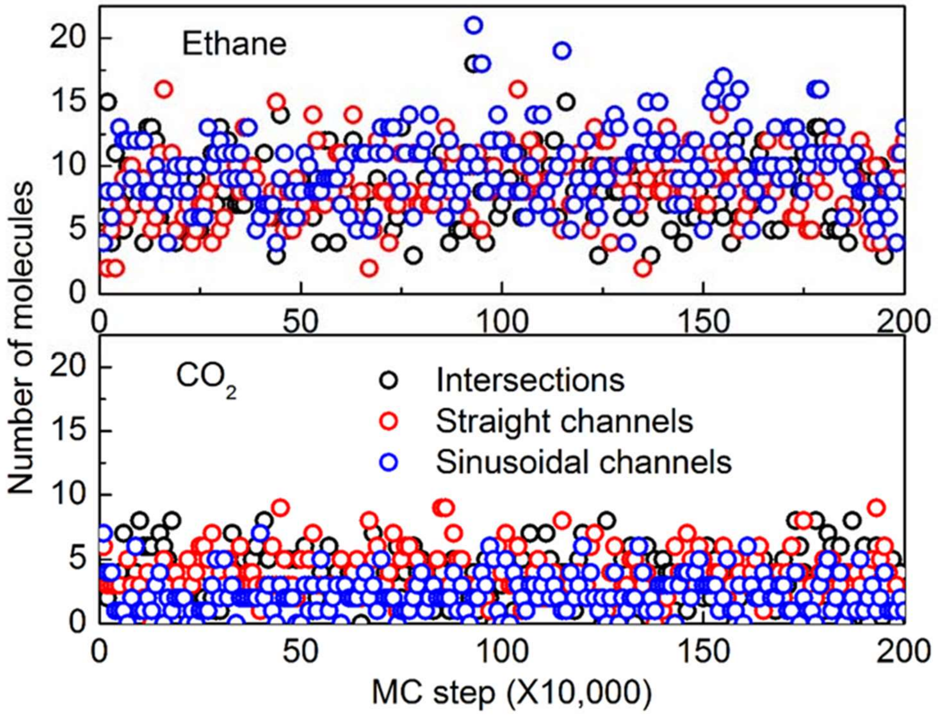

Figure S2. Distribution of ethane and CO2 in straight and sinusoidal channels and their intersections at 0.05 bar. Even though the total number of sorbed molecules at this pressure is low, all types of pores get populated.

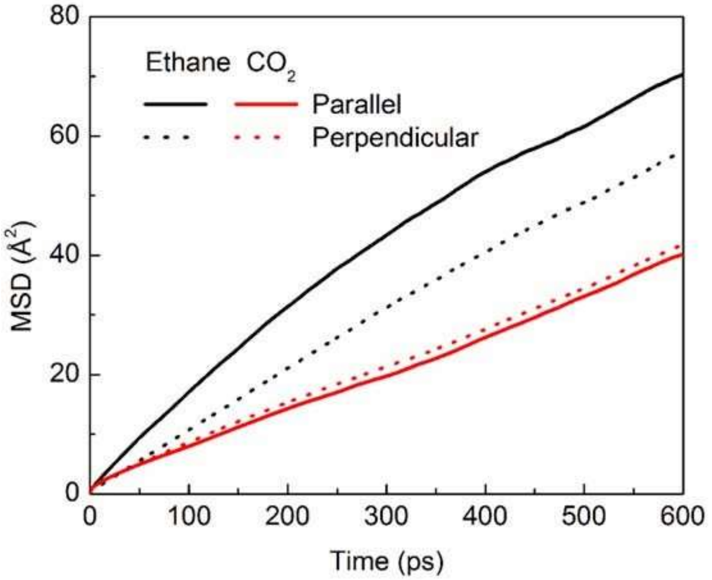

Figure S3. MSD of ethane and CO_2 parallelel and perpendicular to the molecular axis at 100 bar. Note that the MSD in a direction perpendicular to the molecular axis shown here has been divided by 2 for normalization. This is because, while only one direction can be parallel to the molecular axis, 2 mutually orthogonal directions can be perpendicular to the molecular axis.
